# Editorial for the Special Issue: Advances in the Biology of Phototrophic Bacteria

**DOI:** 10.3390/microorganisms9102119

**Published:** 2021-10-08

**Authors:** Johannes F. Imhoff

**Affiliations:** GEOMAR Helmholtz Centre for Ocean Research, D-24105 Kiel, Germany; jimhoff@geomar.de

Phototrophic bacteria represent a very ancient phylogenetic and highly diverse metabolic type of bacteria that diverged early into several major phylogenetic lineages with quite different properties. First of all, they differ in the structure of the photosynthetic apparatus, the light reactions, bacteriochlorophyll structure and biosynthesis. They are different in the relation to oxygen, i.e., are oxygen producers, are strict anaerobes or have a wide range of tolerance of oxygen. Some species can make use of oxygen for energy generation. They have adapted to all kinds of ecological niches and representatives are found in a wide range of environments from cold waters to hot springs, from freshwater to saturated salt brines, from acidic to alkaline habitats, in microbial mats and as inhabitants inside rocks. Among a number of books on phototrophic bacteria, two compendia that highlight major topics of early studies shall be mentioned [1,2].

Over the past decades, genomic and transcriptomic studies have pushed our knowledge on phototrophic bacteria in various aspects, especially in regard to phylogenetic relationships of species and evolution of physiological pathways, but also in the analysis of environmental communities and the metabolic flexibility and adaptation of communities and individual strains to specific ecological niches and to changing environmental conditions.

This Special Issue highlights recent advances in these aspects. It includes results specifically on green sulfur bacteria (*Chlorobi* [3]), heliobacteria (*Firmicutes* [4]), *Chloroflexi* [5], *Cyanobacteria* [6,7] and phototrophic purple bacteria (*Proteobacteria* [8,9,10,11,12,13]). In addition, phylogenetic studies regarding photosynthesis and osmotic adaptation consider representatives of all of these phyla [14,15]. This Special Issue includes results on identification and genomic characterization of new isolates [9], the adaptation to specific ecological niches in regard to temperature [6], salinity [14] and pH [4]. It also considers aerobic phototrophic purple bacteria and the relations to oxygen and oxidative stress [8,9,10,11,13] as well as environmental interactions [5,7,12] and syntrophic relations [3].

Photosynthesis is a key process in the development of life on earth, and over roughly 3.5 billion years of phototrophic life on earth, not only a number of significantly different phylogenetic lineages diverged but also different ecological niches were conquered. The analysis of gene sequences of a key enzyme in bacteriochlorophyll biosynthesis, the light-independent chlorophyllide reductase BchXYZ which is common to all anoxygenic phototrophic bacteria, including those with a type-I and those with a type-II photosynthetic reaction center, highlights their phylogenetic relationship [15]. The phylogenetic relations of more than 150 species demonstrate that bacteriochlorophyll biosynthesis had evolved in ancestors of phototrophic green bacteria (*Chlorobi, Heliobacteriaceae*, *Chloracidobacterium*) much earlier as compared to phototrophic purple bacteria and also that multiple events independently formed different lineages of aerobic phototrophic purple bacteria, some of which have ancient roots [15].

Apparently, phylogenetically distinct strains of the *Chlorobi* have specifically adapted to form syntrophic associations [16,17]. It has been the careful analysis of a mixed culture considered to be a green sulfur bacterium and named “Chloropseudomonas ethylica” that led to the discovery of a syntrophic relationship of a green sulfur bacterium and a chemotrophic sulfur-reducer realizing a short type of sulfur cycle [17]. Now, the simultaneous determination of the genome sequences of the green and colorless components of three mixtures named “Chloropseudomonas ethylica” N3 and N2 originating from E.N. Kondrat’eva many years ago and of the 2-K mixture (DSM 1685) revealed the identity of the green component as a distinct *Prosthecochloris* species and of the colorless component as a distinct cluster within *Desulfuromonas* strains [3]. *Prosthecochloris ethylica* is proposed as a new species name for the green component. Tight adhesion (Tad) types of pili are suggested to play a role in the syntrophic relationship of these bacteria by forming cell–cell interactions and a gene cluster encoding these pili is characteristic for those green sulfur bacteria (N2, N3, and DSM1685) that are involved in the interactions [3]. The formation of pili in both partners of the syntrophic association suggests an evolutionarily gained specific property of the syntrophic partners [3].

Over evolutionary times, phototrophic bacteria that have conquered different ecological niches have given rise to the evolution of phylogenetic groups of species that are living today in these niches. This is depicted in specific ecological niches of phylogenetic distinct groups of phototrophic bacteria with examples of some heliobacteria in alkaline waters [4], of different groups of phototrophic purple bacteria adapted to marine and hypersaline habitats [14] and of certain genera of cyanobacteria with different temperature responses [6]. The last aspect was studied by comparison of temperature responses of 30 isolates of these genera and by a meta-analysis of 84 locations around the world [6] and revealed advantages of *Tolypothrix* strains at lower temperatures, *Scytonema* strains at higher temperatures and *Nostoc* strains at moderate temperatures [6]. The complex situation in the habitat was demonstrated by an expanded upper temperature range for growth if fixed nitrogen sources are available.

Alkaliphilic heliobacteria of the genus *Heliorestis* live in soda lakes, grow optimally between pH 8.0 and 9.5 and form a phylogenetic distinct group among heliobacteria [4]. One of these, *Hrs. convoluta* is the first heliobacterium isolated from a soda lake in the Wadi-el-Natrun (Egypt), which is known as a habitat of alkaliphilic and extremely halophilic eubacteria and archaea, e.g., *Halorhodospira* and *Natronomonas* species [18,19,20]. The analysis of the complete genome sequence of *Hrs. convoluta* provided insight into the molecular adaptation to alkaline conditions, the photoheterotrophic metabolism, nitrogen utilization, sulfur assimilation, and pigment biosynthesis pathways of heliobacteria [4]. Recent genome analyses of a larger number of heliobacteria have led to reconsidering their phylogeny and systematic treatment and confirmed the distinct phylogenetic position of the alkaliphilic *Heliorestis* species [21].

Comparative genomic analyses have consolidated the phylogenetic relationships of phototrophic bacteria living in marine and hypersaline environments. Halophilic phototrophic bacteria have glycine betaine and ectoine as major compatible solutes [22,23] and their ability to transport and synthesize these compatible solutes has been found to correlate well with the occurrence of these bacteria in saline and hypersaline habitats [14]. Furthermore, phylogenetic relations of key genes of these pathways define different phylogenetic groups of halophilic phototrophic bacteria [14].

Another property that correlates with the occurrence in marine or freshwater habitats is the formation of siderophores by aerobic phototrophic bacteria [8]. As important iron chelators, siderophores participate in chelating and uptake of iron from the environment. Interestingly, a high proportion of siderophore producers among aerobic phototrophic purple bacteria was found in isolates originating from freshwater sources (hot springs, freshwater lakes, and biological soil crusts) in comparison to those from marine, meromictic lake, and saline spring habitats [8]. Halotolerant or halophilic aerobic phototrophic purple bacteria do not produce siderophores of equal activity or quantity as compared to bacteria that do not depend on NaCl for growth [8].

A special ecological niche of cyanobacteria as the primary settlers is the endolithic habitat. In this study, the primary colonization and following succession dynamics in intertidal carbonate rocks were studied over a period of nine months [7]. Based on 16S rRNA gene libraries, an “unknown boring cluster” of so far uncultured cyanobacteria was identified as the dominant primary settler [7]. With time, these primary settlers were replaced by other endolithic cyanobacteria and significant populations of anoxygenic *Chloroflexi* occurred in the mature endolithic intertidal ecosystem [7].

Prior to the establishment of oxygenic photosynthesis, phototrophic life had depended on anoxic and strongly reducing conditions. The appearance of oxygen primarily caused severe stress on all strictly anaerobes and triggered adaptation processes to tolerate and eventually use oxygen for energy generation. Today, for many phototrophic purple bacteria, the chemocline and boundary between oxic and anoxic/sulfidic parts of the environment offer conditions for significant developments if light is available. Different strategies have been realized to adapt to these dynamic ecological niches.

The relations to sulfide and oxygen remain important environmental factors determining the distribution and competition of anaerobic phototrophic purple bacteria in the environment. While purple sulfur bacteria have a preference for anoxic and sulfidic parts of the gradients, purple nonsulfur bacteria, to a different degree, have adapted to less sulfidic/sulfide-free, anoxic, microoxic or even oxic parts. In colored blooms and microbial mats, phototrophic purple nonsulfur bacteria and purple sulfur bacteria regularly occur together with a clear preference for the purple sulfur bacteria in sulfidic niches. The different niches of phototrophic purple bacteria were highlighted in a comparison of colored blooms in a coastal environment and in wastewater ditches [12]. The sulfidic coastal marine habitat contained purple sulfur bacteria as the major populations, and smaller but significant densities of purple nonsulfur bacteria, with members of *Rhodovulum* predominating. The freshwater/wastewater habitat exclusively yielded purple nonsulfur bacteria, with species of *Rhodobacter*, *Rhodopseudomonas*, and/or *Pararhodospirillum* as the major constituents. As important environmental factors affecting purple nonsulfur bacteria populations, organic matter, sulfide concentrations and the oxidation-reduction potential were identified [12]. Light-exposed, sulfide-deficient water bodies with high content of organic matter and within a limited range of oxidation-reduction potentials provide favorable conditions for the significant growth of purple nonsulfur bacteria [12].

Many phototrophic purple bacteria that perform photosynthesis under anoxic conditions have alternative ways of energy generation in the dark. The relation to oxygen is a crucial point in their life and oxygen is expected to determine the metabolic activities of these bacteria. They can switch between aerobic and anaerobic lifestyles and responses to these changes are regulated by a complex network of regulators.

*Rba. sphaeroides* and *Rba. capsulatus* are two of these facultative phototrophic bacteria which are able to adjust their lifestyle. They can perform photosynthesis under anoxic conditions but can also perform aerobic or anaerobic respiration or fermentation. Oxygen is a major regulatory factor for the formation of photosynthetic complexes, and several proteins involved in oxygen-mediated gene regulation have been identified [11]. In the presence of light and oxygen, they may be exposed to photooxidative stress by the formation of reactive singlet oxygen. In this study, the responses to photooxidative stress of *Rba. sphaeroides* and *Rba. capsulatus* are compared by transcriptomic and proteomic analyses. Although both species have quite a similar lifestyle, they show different responses to photooxidative stress [11].

Functional iron–sulfur clusters have diverse and important functions in phototrophic bacteria. They are essential for bacteriochlorophyll synthesis and photosynthetic electron transport and are involved in the defense of oxidative stress. A complex regulatory network of several promoters and regulatory proteins is supposed to adjust iron–sulfur cluster assembly to changing conditions in *Rba. sphaeroides* to avoid destruction by oxidative stress [13]. A model is proposed for the regulation of iron–sulfur cluster biosynthesis in which IscR is the main regulator and multiple promotors and regulators are involved in adjusting *ics-suf* expression to environmental conditions [13].

Another study with two facultative phototrophic bacteria, *Dinoroseobacter shibae* and *Rba. capsulatus*, demonstrates that adaptation processes to changes between oxic and anoxic conditions are controlled by a complex regulatory network with different transcriptional regulators in the two species [10]. It is shown that regulation of the CtrA regulon in *Dinoroseobacter shibae* is controlled by the aerobic-anaerobic regulators Crp/Fnr and by FnrL/RegA in *Rba. capsulatus* [10].

An increasing number of phototrophic purple bacteria have been recently identified to be well adapted to the oxic environment. A new isolate, closely related to *Sphingomonas glacialis* and *Sphingomonas paucimobilis*, is characterized on the basis of its complete genome with detailed phenotypic, phylogenetic, genomic, physiological, and biochemical characterization [9].

Counteracting activities of oxygenic photosynthesis and respiration cause changes in stratified environments with moving oxygen horizons during the daily cycles. These are most pronounced in microbial mats. Bacteria living in these gradients have to deal with these regular changes. Prominent examples of bacteria adapted to microbial mats in hot springs are the *Chloroflexus* species [24]. The changes between oxic and anoxic conditions as well as between light and dark conditions force these gliding bacteria to move up and down in the mat to meet optimal conditions and to change key metabolic reactions to optimize energy generation under the variable conditions. A particularly detailed study on the adaptation to these dynamic environmental conditions, especially to daily moving gradients in microbial mats, has been performed with *Chloroflexus aggregatus* by metatranscriptomic and proteomic studies [5], in which the in situ metabolic activity of *Chloroflexus aggregans* in microbial mats of Nakabusa Hot Springs was analyzed [5]. This study reveals a well-coordinated regulation of key metabolic processes to assure that *Cfl. aggregans* uses its metabolic flexibility and capability for both phototrophic and chemotrophic growth to optimize its performance under the varying environmental conditions in its natural habitat [5]. During daytime, light is the main energy source supporting phototrophic growth of *Cfl. aggregans*. During the afternoon, under microoxic low-light conditions, chemoheterotrophic growth is based on aerobic respiration, while fermentation takes place under anoxic conditions at night. During early morning hours before sunrise, chemoautotrophic growth with oxygen as the terminal electron acceptor takes place [5]. During the daily cycle, *Cfl. aggregans* obviously also makes use of both forms of the Mg-protoporphyrin monomethylester cyclase to synthesize bacteriochlorophyll via the aerobic AcsF-dependent enzyme and via the anaerobic BchlE-dependent enzyme [5].

Altogether, the papers of this Special Issue demonstrate the exiting advances that have been made in our knowledge on various aspects of the biology of phototrophic bacteria by the application of genomic, transcriptomic and proteomic analyses. They bring new dimensions to our understanding of metabolic processes and their regulation and to environmental adaptation and place these into a phylogenetic context.
